# The Persulphate of Iron as an Agent for the Destruction of Polypus of the Ear

**Published:** 1862-05

**Authors:** E. L. Holmes

**Affiliations:** Chicago


### THE PERSULPHATE OF IRON

### AS AN AGENT FOR THE DESTRUCTION OF POLYPUS OF THE EAR.

By E. L. HOLMES, M. D., of Chicago.

Any one who has had much experience in the treatment of Polypi in the external meatus of the ear, is aware that, while in some cases their entire removal is attended with comparatively little difficulty, in others, a radical cure is not easily effected.

Polypi can sometimes be eradicated by means of a loop of fine wire, passed round them near their attachment to the walls of the meatus. But it is often difficult to adjust the loop in such a manner as to secure the entire removal of the growth, especially where the pedicle is broad and where its exact situation cannot be ascertained.

Instead of the wire loop, we prefer, where instrumental interference is indicated, the use of the wood forceps or the ring forceps of Toynbee. In favorable cases either of these instruments can be passed a suitable distance into the meatus and the polypus seized and extracted. These tumors are liable, however, to return, unless the small portion, which generally remains adhering to the lining membrane of the meatus, is wholly destroyed by astringents or caustics.

Many attempts have been made to destroy polypi of the ear with caustic agents, and several authors report cases in which they have been successful in their use. Toynbee relates the history of several cases in which he removed the common "Rasberry Polypus" by the use of Potassa Cum Calce carefully applied to the exposed surface of the tumor, the process being repeated daily, till the whole structure was destroyed. The same author also states that the small red vascular polypi, which usually grow from the upper part of the meatus near the membrana tympani in young children, and which are rarely larger than a small pea, may be destroyed by milder astringents, such as Liquor Plumbi Subacetatis, Zinc. Sulph, and Tannic Acid. He believes, however, that the removal of the fibro-gelatinous polypi cannot be effected by means of caustics, since their structure is so firm as to resist the action of these applications.

After reading the report of one or two cases, in which nasal polypi had been destroyed by some of the salts of iron, I was induced, in two cases of polypi of the ear to try the effects of Persulphate of Iron, now so much used as a styptic.

The first case was that of a lady about thirty-two years of age. No satisfactory account of the origin of the polypus could be obtained, for, although the patient had been for a long time troubled with dullness of hearing and a disagreeable discharge from the ear, the polypus had not been detected till some time after the appearance of these symptoms. An attempt had been made to remove the tumor, but it soon returned with symptoms much aggravated. Six months after the operation she came under my care much worn and debilitated by pain and loss of sleep. The swelling and tenderness below the ear were so great as to render all motion of the lower jaw impossible. The polypus almost entirely filled the external meatus, and presented the smooth glistening appearance of an ordinary tumor of the same species. There was so much nervous excitement, and the ear was so sensitive to the least touch that it was thought best to administer chloroform in order to make as thorough an examination as possible. On introducing a probe along the lower surface of the meatus, about half an inch, the bone was found denuded and roughened. I succeeded in passing a loop around the tumor and extracted a large portion of it, removing as much as possible of the remainder with a pair of forceps. On the next day the symptoms were the same, except that the pain was less. But at the end of a fortnight the tumor had returned to nearly half its former size. I had made use of of instruments in this case because I feared the Persulphate of Iron might destroy the tissues around the ulcerated portions of the meatus, and thus produce a larger necrosis. I therefore determined to use it in the following manner: I prepared small cylinders of the sault about a quarter of an inck in length and scarcely a line in diameter, from a thick paste of the iron and mucilage well rubbed together. Into a puncture, half an inch deep, which I made in the tumor with a narrow knife, I introduced one of these cylinders. On the next day the pain had not increased, On syringing the ear gently with warm water, and drying the passage with tufts of cotton, I found the meatus closed with a hard gray mass, which, on removal, proved to be a large portion of the polypus firmly coagulated. Another cylinder was introduced in the same way the next day with the same result. Subsequently I merely applied small quantities of the salt to the polypus, keeping it in place with a bit of cotton pressed firmly against it. This was repeated daily, the quantity of iron being decreased as the deeper portion of the polypus was reached. This was soon completely destroyed, and in a week or ten days two small lamina of bone exfoliated, and the ulcerated surface of bone healed. At the present time, three months since, the termination of treatment, there has been no evidence of the return of the polypus; but the hearing is almost totally lost, and the jaw is still somewhat confined in its movements. The health of the patient, however, is completely restored.

The second case in wich I have used the Persulphate of Iron was that of a girl, thirteen years of age. The presence of the polypus had not been detected till a short period previous to visiting me for the first time. It was a glistening fibro-cellular growth, quite firm in consistence, extending nearly to the external opening of the meatus. Twenty-four hours after the introduction of a cylinder of the salt, as above described, I removed a large piece of the polypus, firmly coagulated. I afterwards applied the dry powder, daily, in small quantities, to the tumor for a week, at the end of which time, it was wholy destroyed. Quite a small red surface near the membrana tympani, on the posterior and lower wall of the meatus, probably the place of attachment of the growth, remained covered with granulations; but these disappeared in a few days under the influence of the iron. In neither of these cases did the salt produce much pain; except from one or two applications there was *no* pain caused by the treatment. After applying this salt several times to mucous membranes and to surfaces covered with the most delicate cuticle, I am disposed to believe that there is little danger of injury to the adjacent tissues, either in polypus of the ear, nose, or of the uterus, when applied with care.

A more extended experience will be necessary to establish the absolute value of this agent in the destruction of polypoid growths.

